# FSH Stimulation with Short Withdrawal Improves Oocyte Competence in Italian Mediterranean Buffalo (*Bubalus bubalis*)

**DOI:** 10.3390/ani10111997

**Published:** 2020-10-30

**Authors:** Georgios Petrovas, Michal Andrzej Kosior, Giorgio Antonio Presicce, Marco Russo, Gianluigi Zullo, Giuseppe Albero, Serhat Alkan, Bianca Gasparrini

**Affiliations:** 1 Veterinary Faculty, Istanbul University-Cerrahpasa, 34320 Istanbul, Turkey; giorgospetrovas@gmail.com (G.P.); salkan@istanbul.edu.tr (S.A.); 2 Department of Veterinary Medicine and Animal Production (DMVPA), Federico II University of Naples, 80137 Naples, Italy; michalandrzej.kosior@unina.it (M.A.K.); g.zullo@anasb.it (G.Z.); giuseppe.albero@unina.it (G.A.); bgasparr@unina.it (B.G.); 3 ARSIAL, Centro Sperimentale per la Zootecnia, 00162 Rome, Italy; g.presicce@arsial.it; 4 National Buffalo Breeders’ Association (ANASB), 81100 Caserta, Italy

**Keywords:** buffalo, OPU, IVEP, coasting, embryo

## Abstract

**Simple Summary:**

The high embryo production cost in buffalo, compared to cattle, is currently the major factor limiting the commercial application of ovum pick-up and in vitro embryo production technologies. This is mainly due to the lower number of follicles that develop during each estrous cycle and consequently oocytes recovered per session. In this work, we tested two different hormonal treatments based on commercial follicle-stimulating hormone (FSH) and progesterone (P4) to enhance the number of follicles and competent oocytes. Furthermore, in the second experiment, we tested three different coasting times, i.e., periods between the last FSH administration and ovarian aspiration, which is known be the key period for the final oocyte maturation and competence acquisition. The results, in terms of number of aspirated follicles, oocyte quality, blastocyst yield, and superior quality blastocyst yield, allow us to suggest the use of high doses of FSH (40 mg given six times every 12 h) combined with the shorter coasting time (28–32 h) as the ideal protocol for ovarian superstimulation in buffalo.

**Abstract:**

The aim of this work was to evaluate the efficiency of different FSH doses and FSH coasting times before ovum pick-up (OPU) on follicular growth and oocyte competence in buffalo. Experiment 1 involved two different FSH treatments: 40 mg FSH given three (FSH3) or six (FSH6) times, 2 days after dominant follicle removal were tested, with OPU carried out after 40–44 h of coasting. In experiment 2, OPU was carried out after FSH6 protocol followed by 28–32 h (C1), 40–44 h (C2), or 64–68 h (C3) of coasting time. Cumulus oocyte complexes (COCs) were classified, in vitro matured, fertilized, and cultured. The results demonstrated that FSH6 increased the total number of follicles, the number and percentages of medium and large follicles, the number and the proportion of good quality oocytes, and the number of grade 1,2 and fast-developing blastocysts compared to the control. C3 decreased the percentage of good quality oocyte and blastocyst rates compared to C1 and C2. A higher percentage of fast blastocysts and average number of grade 1,2 blastocysts was observed in C1 compared to C3, with intermediate values found in C2. The improved efficiency in terms of blastocyst yields suggests the use of FSH6 + C1 protocol for ovarian superstimulation in buffalo.

## 1. Introduction

Buffalo is the livestock species that has undergone the greatest increase in number of heads, milk production, and economical importance in the last decades [1,2]. The Italian Mediterranean buffalo is greatly requested around the world because of its high milk production, and it is the only breed with its own studbook. The possibility of accelerating genetic development in buffalo (*Bubalus bubalis*) is linked to the use of reproductive technologies, such as artificial insemination (AI), multiple ovulation and embryo transfer (MOET), and ovum pick-up (OPU) combined with in vitro embryo production (IVEP). Unlike in cattle, MOET is not commercially feasible in water buffalo because of the low number of embryos recovered per session, likely due to a defect in the capture of the oocyte by the fimbria and its transport through the oviduct [3,4,5]. Therefore, OPU is undoubtedly the best tool to rapidly speed up the genetic progress through the maternal lineage in this species [6]. Despite a low number of blastocysts produced per session, OPU-IVEP allows higher embryo yields on a long-term basis, as it can be repeated for long periods without interfering with the reproductive activity of the donors [7,8]. The lower production of blastocysts, compared to cattle, is due to the lower number of follicles developing during each estrous cycle and consequently oocytes recovered per session [6], undoubtedly related to the small reserve of primordial follicles [9,10]. In addition, seasonal effects on oocyte competence have been demonstrated [11,12], further limiting gamete availability in this species. The low oocyte recovery, resulting in high embryo production costs, is certainly the major factor limiting the commercial application of OPU in buffalo. Although donors can be selected by either evaluating the antral follicular count [13] or measuring anti-Mullerian hormone [14,15], this approach often results in the exclusion of high genetic merit buffalo cows from the programs. Several treatments have been used to stimulate follicular growth and improve oocyte competence in cattle [16,17,18,19]. It is known that the size of the follicle is positively associated with oocyte competence in cattle, with the greatest embryo production obtained aspirating oocytes from 6 to 10 mm diameter follicles [20,21,22,23,24]. Furthermore, a period of arrest of gonadotropin support under endogenous luteinizing hormone (LH) is needed for final follicular differentiation and improvement of oocyte competence [19,25,26]. Indeed, follicle-stimulating hormone (FSH) withdrawal before aspiration (*coasting*) leads the oocyte to complete its post-transcriptional RNA modifications and chromosome segregation processes [27] and allows the maximum number of “pseudo-dominant” oocytes to complete the development under basal LH effect.

In buffalo, there is a paucity of data in literature regarding hormonal treatments to enhance the number of follicles and competent oocytes. Recently, two studies on Murrah buffaloes demonstrated the positive effect of different FSH stimulation protocols on oocyte competence [28,29]. However, to our knowledge, no information is available in buffalo, neither regarding the optimal FSH dose nor the optimal coasting time. Interestingly, in non-stimulated donors, although the number of oocytes recovered per session in buffalo is much lower than in cattle, blastocysts yields are similar [29,30,31,32]. In contrast, the improved blastocyst yields recorded in cattle after FSH stimulation is undoubtedly more impressive than that observed in buffalo [20,29,33,34]. This supports the hypothesis that, although the estrous cycle and the hormonal and molecular regulatory mechanisms are similar to cattle, the acquisition of oocyte competence in buffalo can take place in temporally different ways.

Therefore, the aim of this work was to evaluate first the efficacy of FSH stimulation prior to OPU with different doses of FSH in the presence of progesterone in Italian Mediterranean buffalo (experiment 1). A further objective was to assess the effect of different FSH deprivation (coasting) times (experiment 2) in order to identify the ideal *modus operandi* capable of promoting the growth of a large pool of medium-large size follicles and the recovery of competent oocytes.

## 2. Materials and Methods

All chemicals and reagents, if not otherwise stated, were purchased from Sigma (Sigma-Aldrich, Milan, Italy).

### 2.1. Animals

The Ethical Animal Care and Use Committee of the University of Naples Federico II (Naples, Italy) approved the experimental design and animal treatments (PG/2029/007004 of 2 July 2019). In both experiments, multiparous Italian Mediterranean buffalo (*Bubalus bubalis*) cows under controlled nutrition and housed inside barns at a farm located in the province of Caserta (Italy) were enrolled. All animals were assessed before the beginning of the experiments, and only healthy, cyclic buffaloes without any pathologies of the genital tract were selected. In experiment 1, buffaloes (*n* = 18), with average age, parity, days in milk, and milk production of 6.4 ± 0.7 years, 3.5 ± 0.6, 226 ± 57 days, and 2760 ± 11.0 kg, respectively, were used. In experiment 2, buffaloes (*n* = 15) with average age, parity, days in milk, and milk production of 7.8 ± 0.8 years, 4.1 ± 0.6, 115.2 ± 29.7 days, and 3340 ± 17.0 kg, respectively, were enrolled.

### 2.2. Experimental Design

**Figure 1:**
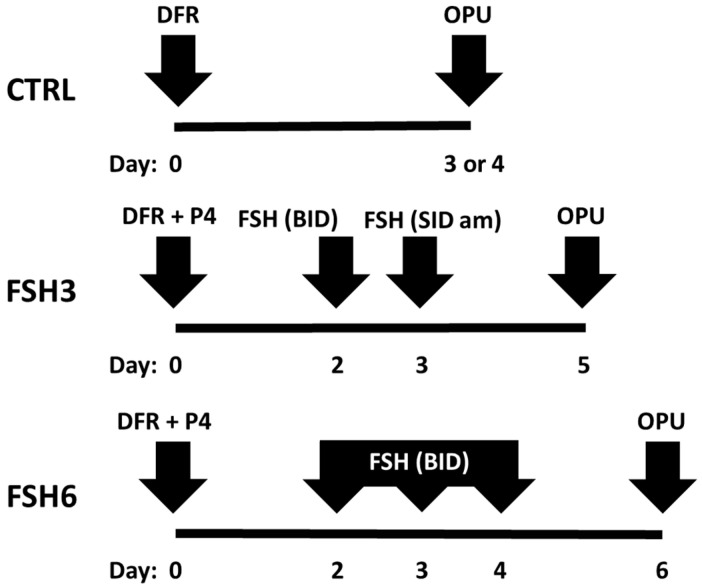
Exemplification of different ovum pick-up (OPU) treatments in experiment 1.

The trial was carried out during the breeding season (September–December). Experiment 1 was carried out to evaluate the effect of two different FSH doses compared to a control, as shown in Figure 1. All buffaloes (*n* = 18) underwent 12 OPU sessions (4 sessions per each treatment)—the first 4 sessions without stimulation (control) and then alternatively after 2 different FSH treatments, i.e., 40 mg every 12 h for 3 times (FSH3 group) or 40 mg every 12 h for 6 times (FSH6 group). Buffaloes underwent dominant follicle removal (DFR) via transvaginal aspiration on day 0, followed by 4 consecutive OPU sessions at a distance of 3–4 days (control group). In treated groups, at the time of DFR (day 0), we used a progesterone (P4)-releasing intravaginal device (PRID Delta 1.55 g, Ceva Animal Health Ltd., Amersham, Buckinghamshire, United Kingdom), then starting on day 2, we administered 120 mg (FSH3 group) or 240 mg (FSH6 group) FSH (Folltropin, Vétoquinol S.A., Magny-Vernois, France), as previously described. In treated groups, OPU was carried out on days 5 and 6 in FSH 3 and FSH 6 groups, respectively (40–44 h of coasting time).

In experiment 2, on the basis of the results of experiment 1, we chose the FSH6 treatment to evaluate the effect of coasting time on oocyte competence. The experiment was carried out on a total number of 15 buffaloes. Animals were treated as previously reported for the FSH6 group and OPU was carried out at different coasting times, i.e., 28–32 h (*n* = 5, C1 group), 40–44 h (*n* = 5, C2 group), and 64–68 h (*n* = 5, C3 group) after the last FSH administration. The animals were randomly exposed to all the 3 coasting times, in a cross-over design. A total number of 6 consecutive OPU sessions was carried out.

### 2.3. Ovum Pick-Up Procedure and COC Processing

Ovum pick-up consisted of the use of a portable ultrasonic unit Sonoace Pico (Medison, Seoul, Korea) with a 5 MHz sector scanner micro-convex probe mounted on a properly designed vaginal support (WTA, Ltd.a, Cravinhos/SP, Brazil) equipped with a guide for 18 gauge needles. A vacuum pressure of 40 mm-Hg was constantly maintained by using a suction unit (K-MAR-5100, Cook IVF Co., Queensland, Australia) and the aspiration line was continuously rinsed with phosphate-buffered saline (PBS; HyClone 1X, GE Heltcare Life Science, South Logan, UT, USA) supplemented with 100 USP units/mL^−1^ of heparin (Eparina Vister, Teva, Italy), 1% fetal calf serum (FCS), and 1% penicillin and streptomycin complex (Pen-Strep; 20,000 IU and 20,000 g/mL, respectively) during follicular aspiration. All visible antral follicles (total follicles, TFL) were punctured and classified into small (SFL, diameter <0.5 cm), medium (MFL, diameter between 0.5 and 1 cm), and large (LFL, diameter >1 cm). The 50 mL conical tube (Falcon, Corning Science, Reynosa, México), containing 5 mL of PBS supplemented as described above, was used to start the oocyte collection, and a second tube full of same solution was used during and at the end of the procedure for cleaning the circuit, with a temperature constantly maintained at 37 °C for both tubes. The aspirated solution containing cumulus oocyte complexes (COCs) was passed through a 70 µm sterile nylon strainer (Corning, Life Science, Durham, NC, USA), which was then washed with PBS solution without Pen-Strep complex in Petri dishes (90 × 15 mm). The COCs were searched immediately, washed twice in N-(2-Hydroxyethyl)piperazine-N′-(2-ethanesulfonic acid) (HEPES)-buffered tissue culture medium (TCM) 199 (H 199) with 10% FCS in a 35 mm Petri dish and classified according to their morphology, as previously described [11], on the basis of the number of compact cumulus investments layers and presence of homogenous cytoplasm. Only grade A + B + C COCs were further processed for IVEP. Once classified, the COCs were transferred in 1.8 mL sterile Cryovials tubes (Simport, Beloeil, QC, Canada) filled to the top with pre-equilibrated maturation medium consisting of H 199 supplemented with 50 µM cysteamine, 0.5 µg/mL FSH, 5 µg/mL LH, and 1 µg/mL 17-β-estradiol, which were transported to the laboratory in a portable incubator (WTA, Cravinhos, SP, Brazil) at 38.7 °C within 4–6 h. For each session, the recovery rate, the number of total cumulus oocyte-complexes (COCs), COCs suitable for IVEP (grade A + B + C), total discarded, and expanded COCs were recorded.

### 2.4. In Vitro Embryo Production

In this trial, the oocytes recovered per each treatment/session were pooled for in vitro maturation (IVM), fertilization (IVF), and culture (IVC), as the benefits of culturing gametes [35] and embryos [36,37] in groups rather than individually on IVEP efficiency are well known. Once COCs-containing tubes reached the laboratory, IVM was completed by transferring COCs into 50 µL droplets (10 COCs/droplet) of the final maturation medium, which had the same composition described above but was buffered with 25 mM of sodium bicarbonate and pre-equilibrated in incubator under mineral oil. The full droplets were incubated at 38.7 °C for 22 h under controlled gas atmosphere of 5% CO_2_ in humidified air. In vitro fertilization was conducted according to the method previously described [38]. To reduce the paternal effect, we used semen from the same bull, which was previously proven suitable for IVF, for both experiments. Frozen–thawed semen was prepared by Percoll density gradient (Nidacon, Mölndal, Sweden). The sperm pellet obtained after centrifugation was re-suspended to a final concentration of 2 × 10^6^/mL in the IVF medium with the following composition: modified TALP supplemented with 0.2 mM/mL penicillamine, 0.1 mM/mL^−1^ hypotaurine, and 0.01 mM/mL heparin. In vitro matured oocytes were removed from the IVM drops, thoroughly washed in IVF medium, allocated in 50 µL fertilizing droplets (5 COCs/droplet) covered by mineral oil, and incubated under the same gas atmosphere as for IVM. After 20–22 h of co-incubation with spermatozoa, presumptive zygotes were stripped of cumulus cells by gentle pipetting and washed twice in H 199. They were then in vitro cultured in 20 µL droplets (10 presumptive zygotes/droplet) of synthetic oviduct fluid (SOF) medium [39] and supplemented with essential and non-essential amino acids and bovine serum albumin (BSA) for 7 days in a modular chamber with a gas atmosphere of 5% CO_2_, 5% O_2_, and 90% N_2_. At day 5 (day 0 = IVF day), cleavage rate was assessed, and embryos were transferred into fresh droplets of the same medium for a further 2 days of culture. The percentages of superior quality blastocysts (grade 1,2 BL) and grade 1,2 fast developing blastocysts (fast BL) were evaluated, out of the total COCs, at day 7 of culture. Furthermore, numbers of grade 1,2 BL and fast BL per donor were recorded.

### 2.5. Statistical Analysis

Differences among groups for both experiment 1 and 2 in the mean numbers of small, medium, large and total aspirated follicles, as well as in the total COCs and grade A + B + C COCs, were analyzed by ANOVA for repeated measures test using SPSS IBM version 22.0 statistical software (IBM Corp. Released 2013. IBM SPSS Statistics for Windows, Version 22.0. Armonk, NY, USA: IBM Corp; 2013). Likewise, recovery, cleavage, and blastocyst production rates, as well as number of grade 1,2 BL and fast BL/donor were analyzed. All the data were presented as means ± standard error (mean ± SE). The level of significance to reject the null hypotheses (H0) was 5%, and a variable was considered significantly different when *p* < 0.05. In addition, the FSH6 group also improved (*p* < 0.01) the number of large follicles compared to the control. The FSH3 increased (*p* < 0.05) the number of small follicles compared to the control, with intermediate values in FSH6. The FSH6 priming also increased the percentage of medium (*p* < 0.01) and large (*p* < 0.05) follicles, while decreasing (*p* < 0.01) the percentage of small follicles compared to the other groups.

## 3. Results

### 3.1. Experiment 1

As shown in Table 1, both priming treatments increased (*p* < 0.01) the number of total and medium follicles compared to the control group; however, FSH6 priming was more effective than FSH3. In addition, the FSH6 group also improved the number of large follicles compared to the control. The FSH3 increased the number of small follicles compared to the control, with intermediate values in FSH6. The FSH6 priming also increased the percentage of medium and large follicles, while decreasing the percentage of small follicles compared to the other groups.

The parameters related to oocyte morphology and competence are also shown in Table 1. The FSH6 priming increased (*p* < 0.01) the mean number of total recovered COCs and grade A + B + C COCs per donor per session compared to both the other groups. Recovery rate was higher (*p* < 0.05) in the FSH6 group in comparison with FSH3 group, but similar to the control. Both FSH priming groups increased (*p* < 0.05) cleavage rate compared to the control. However, despite a tendency to increase at higher FSH doses (*p* = 0.08), the percentages of grade 1,2 BL were not significantly improved. Nevertheless, FSH6 priming improved (*p* < 0.01) the mean number of grade 1,2 BL per donor/session by approximately fivefold. Interestingly, the number of fast BL per donor per session was much higher (*p* < 0.05) in the FSH6 group than in the control.

### 3.2. Experiment 2

As reported in Table 2, the coasting time did not affect the total number of follicles, as well as the number of small, medium, and large follicles. Moreover, the percentages of small, medium, and large follicles did not change among groups.

No differences were also observed in the mean number of total COCs recovered per donor per session, while C3 decreased (*p* < 0.05) the percentage of good quality oocytes (grade A + B + C) and increased (*p* < 0.05) the proportion of total discarded and expanded COCs (Table 2).

As shown in Table 2, no differences were found in cleavage rate among groups. However, blastocyst rates decreased (*p* < 0.01) at the highest coasting time (C3), while no differences were recorded between C1 and C2. However, a higher percentage of fast BL was observed in the C1 group in comparison to the C3 group, with intermediate values found in C2. Likewise, the average number of grade 1,2 BL (*p* < 0.05) and fast BL (*p* = 0.06) increased in C1 compared to C3, with intermediate values in C2.

## 4. Discussion

This study investigated the effect of different FSH doses and coasting times on follicular population and oocyte competence in highly productive Italian Mediterranean buffaloes in an attempt to improve the embryo yields per donor, which is currently still the major limiting factor the diffusion of IVEP technology in this species.

In experiment 1, it was demonstrated that the administration of 40 mg of FSH every 12 h for 6 times (FSH6) prior OPU is effective in improving follicular growth and oocyte competence. Indeed, in addition to an increase of the number of total follicles and COCs, this treatment significantly enhanced both the number and the percentages of medium and large follicles compared to the control (unstimulated buffaloes), which was reflected in higher proportions of good quality oocytes. Consequently, a higher cleavage rate and a tendency to improved percentages of superior quality blastocysts (grade 1,2 BL) were also observed after FSH6. More importantly, the number of both grade 1,2 BL and fast BL per donor increased by approximately 4.5- and 7-fold, respectively, compared to the control. The administration of FSH every 12 h for 3 times (FSH3) also promoted a beneficial effect but to a limited extent. In fact, FSH3 increased the total number of follicles and medium follicles and improved cleavage rate, without improving though the blastocyst production per donor. Therefore, halving the FSH dose (120 mg), in the attempt to reduce the treatment costs, was not as effective as for FSH6 (240 mg).

In an earlier trial, a positive effect on oocyte competence and blastocyst production was observed in Murrah buffaloes, with 200 mg FSH given in decreasing doses in a 2-day protocol, in the presence of estradiol and progesterone [29]. An improvement of follicular population and oocyte competence was also recorded in a previous study in Murrah buffaloes by administering decreasing doses of FSH over 3 days followed by GnRH [28]. In prepuberal Italian Mediterranean buffaloes, treatment with decreasing doses of FSH-LH increased the proportion of medium and large follicles, as well as the number of COCs and maturation competence [40]. In our study, the number of follicles and oocytes recovered was lower than that reported in Murrah buffaloes but in line with previous studies carried out on Italian Mediterranean buffaloes in similar geographical conditions [11,41]. These differences can be attributable to various causes, such as breed, environment, and/or management. It is worth noting that our study was conducted on highly productive buffaloes that, despite the low follicular population, gave high blastocyst yields.

The improved oocyte competence recorded with FSH6 was due to the increased proportion of follicles reaching medium-to-large size, as follicles benefit from an extension of the growth phase under FSH stimulation. Indeed, an effect of the follicular size on oocyte competence was previously demonstrated in buffalo, with oocytes recovered from large follicles exhibiting both an improved capacity to undergo maturation and develop into blastocysts [42,43].

As FSH6 was more effective at forcing a larger population of follicles in the growth phase and at improving oocyte developmental competence, this treatment was used for experiment 2. The results of this experiment demonstrated that the coasting time does not affect the total number of follicles, in agreement with previous studies in cattle [33]. This indicates that the follicular recruitment phase is also inhibited in buffalo by the larger follicles and does not continue when the exogenous FSH supplement ceases. Interestingly, the proportion of large- and medium-sized follicles did not rise at increasing coasting times, in contrast with what has been observed in cattle [33]. In cattle, a similar increase in the proportion of medium and large follicles was recorded at 44 h and 64 h, compared to 20 h coasting, and further growth was recorded at 92 h [33]. The results of our study suggest that a shorter FSH deprivation time is needed in buffalo to obtain a great proportion of medium-to-large follicles. As no further growth was observed at the following times, it is likely that the final follicular growth in this species is accomplished earlier. Furthermore, it was demonstrated that an extension of coasting time to 64–68 h (C3) results in a decrease of the percentage of oocytes morphologically suitable for IVM (A + B + C), together with an increase of the percentage of discarded oocytes, i.e., those not used for embryo production. In particular, the majority of the discarded oocytes were expanded and the proportion of this category of oocytes increased at higher coasting times. It is worth mentioning that these oocytes showed abnormally expanded and clustered cumulus cells, resembling the features of degenerating oocytes [11]. The worst oocyte quality recorded in C3 group is reflected by the lower blastocyst yields. No differences in grade 1,2 BL rates were detected between the short (C1) and medium (C2) coasting times, but in C1 there was an improvement of the proportion of fast BL compared to C3, with intermediate values in C2. This is an important parameter, as fast-developing blastocysts have been previously demonstrated to give higher pregnancy rates after embryo transfer in buffalo [44]. Likewise, in C1, the number of both grade 1,2 BL (*p* < 0.05) and fast BL (*p* = 0.06) per donor was approximately three times higher than in C3, with C2 showing intermediate results. This suggests that a shorter coasting time may be more beneficial in buffalo. It is interesting to note that a decreased proportion of good quality oocytes, with an increased proportion of expanded/degenerating oocytes, resulting in reduced blastocyst production, was observed as early as at 64–68 h coasting time, whereas a noticeable decrease in blastocyst yields was reported in cattle only when coasting was prolonged to 92 h [33]. This suggests that there may be a quicker acquisition of competence by the oocyte in buffalo, which is in agreement with the phenomenon of early oocyte aging previously described in this species [45]. Indeed, it was demonstrated that buffalo oocytes undergo aging prematurely, as indicated by worsening of both the morphology and developmental competence after in vitro fertilization and parthenogenetic activation at increasing maturation times [45,46].

## 5. Conclusions

In conclusion, we demonstrated that treatment with FSH improves oocyte competence and embryo production in buffalo; in particular, the administration of 40 mg of FSH twice daily for 3 days increased the number of good quality blastocysts per donor by approximately five times. Furthermore, it was demonstrated that the acquisition of oocyte developmental competence requires a shorter coasting time in buffalo than in cattle. The improved efficiency in terms of blastocyst yields obtained with FSH6 priming with 28–32 h coasting time may facilitate the commercial diffusion of IVEP technology in this species. Further studies are needed to evaluate whether the improved oocyte competence is also associated to higher pregnancy rates after ET.

## Figures and Tables

**Table 1:** Effect of follicle-stimulating hormone (FSH) priming with either three doses (FSH3) or six doses (FSH6) on follicular population, oocyte quality, and developmental competence parameters compared to control.

Parameters	Control	FSH3	FSH6
	Mean ± SE	Mean ± SE	Mean ± SE
Total follicles *n* *	3.4 ± 0.2 ^A^	5.4 ± 0.5 ^B^	7.2 ± 0.5 ^B^
Large follicles *n* * %	0.2 ± 0.1 ^A^ 5.8 ± 2.6 ^a^	0.3 ± 0.1 ^A,B^ 5.1 ± 3.4 ^a^	1.2 ± 0.2 ^B^ 17.1 ± 2.5 ^b^
Medium follicles *n* * %	0.7 ± 0.1 ^A^ 21.1 ± 3.3 ^A^	1.3 ± 0.2 ^B^ 24.1 ± 4.4 ^A^	2.7 ± 0.3 ^B^ 37.4 ± 3.3 ^B^
Small follicles *n* * %	2.5 ± 0.2^a^ 73.1 ± 4.2 ^A^	3.9 ± 0.4 ^b^ 70.8 ± 4.9 ^A^	3.3 ± 0.4 ^a,b^ 45.5 ± 4.0 ^B^
Recovery rate %	54.2 ± 4.6 ^A,B^	41.6 ± 5.2 ^A^	59.5 ± 4.1 ^B^
COCs *n* *	2.3 ± 0.3 ^A^	2.4 ± 0.4 ^A^	4.6 ± 0.5 ^B^
A + B + C COCs *n* * (%)	1.3 ± 0.2 ^A^ (56.5 ± 4.9) ^A,a^	1.7 ± 0.3 ^A^ (69.3 ± 6.8) ^b^	3.5 ± 0.4 ^B^ (75.7 ± 4.1) ^B^
Cleavage *n* * (%)	0.6 ± 0.1 ^a^ (34.3 ± 3.8) ^a^	1.0 ± 0.1 ^a^ (43.4 ± 0.2) ^b^	2.1 ± 0.2 ^b^ (51.5 ± 2.3) ^b^
Grade 1,2 BL *n* * (%)	0.2 ± 0.1 ^A^ (10.3 ± 7.0)	0.4 ± 0.3 ^A^ (18.8 ± 12.0)	0.9 ± 0.1 ^B^ (23.6 ± 2.4)
Fast BL *n* * (%)	0.1 ± 0.1 ^a^ (8.0 ± 6.5)	0.4 ± 0.2 ^a,b^ (16.2 ± 9.5)	0.7 ± 0.1 ^b^ (16.5 ± 2.3)

^A,B^ Values with different superscripts within rows are significantly different; *p* < 0.01. ^a,b^ Values with different superscripts within rows are significantly different; *p* < 0.05. * mean number/donor/session.

**Table 2:** Effect of 28–32 h (C1), 40–44 h (C2), and 64–68 h (C3) coasting times on follicular population, oocyte quality, and developmental competence parameters.

Parameters	Coasting Time		
	C1	C2	C3
	Mean ± SE	Mean ± SE	Mean ± SE
Total follicles *n* *	6.4 ± 0.7	5.2 ± 0.5	6.2 ± 0.6
Large follicles *n* * %	1.6 ± 0.3 29.3 ± 5.1	1.1 ± 0.2 25.0 ± 5.1	1.5 ± 0.3 29.1 ± 5.4
Medium follicles *n* * %	2.3 ± 0.6 30.8 ± 5.0	1.6 ± 0.4 25.6 ± 5.4	2.2 ± 0.4 32.3 ± 4.8
Small follicles *n* * %	2.5 ± 0.4 39.9 ± 5.3	2.5 ± 0.3 49.4 ± 5.1	2.5 ± 0.5 38.6 ± 5.1
COCs *n* *	3.8 ± 0.6	3.5 ± 0.5	4.0 ± 0.7
Recovery rate %	56.0 ± 6.1	66.4 ± 6.2	63.8 ± 6.7
Grade A + B + C COCs *n* * (%)	2.7 ± 0.4 (79.0 ± 5.2) ^a^	2.7 ± 0.4 (78.9 ± 6.5) ^a^	2.2 ± 0.4 (58.6 ± 7.3) ^b^
Expanded COCs *n* * (%)	0.3 ± 0.1 (6.1 ± 3.0) ^a^	0.5 ± 0.2 (14.7 ± 6.4) ^a,b^	1.0 ± 0.2 (26.3 ± 3.3) ^b^
Total discarded COCs *n* * (%)	1.1 ± 0.4 (21.0 ± 5.2) ^a^	0.8 ± 0.2 (21.1 ± 6.5) ^a^	1.7 ± 0.4 (41.4 ± 7.3) ^b^
Cleavage *n* * (%)	2.4 ± 0.2 (73.8 ± 12.4)	2.0 ± 0.1 (72.7 ± 8.4)	1.0 ± 0.5 (72.8 ± 9.2)
Grade 1,2 BL *n* * (%)	1.4 ± 0.3 ^A^ (41.8 ± 11.1) ^B^	1.0 ± 0.1 ^A,B^ (35.7 ± 2.9) ^B^	0.4 ± 0.1 ^B^ (11.2 ± 3.8) ^A^
Fast BL *n* * (%)	1.2 ± 0.3 (34.8 ± 9.9) ^a^	0.8 ± 0.7 (29.2 ± 1.7) ^a,b^	0.4 ± 0.7 (11.2 ± 3.8) ^b^

^A,B^ Values with different superscripts within rows are significantly different; *p* < 0.01. ^a,b^ Values with different superscripts within rows are significantly different; *p* < 0.05. * Mean number/donor/session.
